# Deciphering carbon source–sink dynamics in masting tree species using tree-ring isotopes

**DOI:** 10.1093/treephys/tpae026

**Published:** 2024-02-24

**Authors:** Jesús Julio Camarero, Ester González de Andrés

**Affiliations:** Department of Ecosystem Conservation, Instituto Pirenaico de Ecología (IPE-CSIC), Avda Montañana 10005, Zaragoza 50009, Spain; Department of Ecosystem Conservation, Instituto Pirenaico de Ecología (IPE-CSIC), Avda Montañana 10005, Zaragoza 50009, Spain

**Keywords:** carbon allocation, drought stress, radial growth, reproductive effort, source-sink dynamics, tree-ring stable isotopes

This scientific commentary refers to ‘Tree-ring isotopic imprints on time-series of reproductive effort indicate warming-induced co-limitation by sink and source processes in stone pine’ by Shestakova et al. (doi:10.1093/treephys/tpad147).

Climate change is leading to warmer conditions and subsequent increases in atmospheric water demand, compounded in many cases by reductions in soil water availability. Consequently, the importance of drought stress on forest dynamics and functioning has intensified in recent decades (Seidl et al. 2017). Climatic projections foresee an upward rise in temperature and increasing aridification in many regions (IPCC 2021), whereby climate change-induced forest disturbances are expected to be significantly exacerbated (Brodribb et al. 2020). Forest ecosystem responses comprise reductions of primary and/or secondary growth (Adams et al. 2015) and drought-triggered mortality episodes (Hammond et al. 2022), and eventually threaten long-term population persistence (Aitken et al. 2008).

Growth and reproduction, two key components of fitness and forest functioning, are two of the main carbon sinks in trees (Körner 2003). The alteration of carbon balance of trees following drought events has been previously reported (Galiano et al. 2011, Piper et al. 2017). Therefore, elucidating the responses of source–sink dynamics is crucial to better understand the adaptative potential of trees and forests in the face of climate change (Dusenge et al. 2019), and to improve the representation of carbon allocation in process-based models of forest functioning. Nevertheless, there is an ongoing scientific debate as to whether growth and reproduction are sink- or source-limited (Sala et al. 2012, Palacio et al. 2014, Körner 2015, Wiley et al. 2017, Mund et al. 2020). Evidence has been found for both processes, since previous research has reported negative (Knops et al. 2007, Hacket-Pain et al. 2018, Rosati et al. 2018) and positive (Garcia-Barreda et al. 2021, Shestakova et al. 2021) relationships between growth and reproductive performance. However, sink limitation has received greater support, as cambium activity has been found to be more sensitive to environmental factors than photosynthesis (Körner 2015, Delpierre et al. 2016), and non-structural carbohydrates reserves of tree tissues are generally high (Klein et al. 2014, Martínez-Vilalta et al. 2016). As for source limitation, it does not necessarily imply low carbon content, but rather allocation to storage to promote osmoregulation or avoid carbon starvation under drought (Sala et al. 2012). Moreover, sink and source limitations are not mutually exclusive, but intermediate situations of co-limitation may occur, their spatiotemporal dynamics being modulated by prevailing environmental conditions (Ainsworth et al. 2004). In addition, negative correlations between radial growth and seed production do not imply trade-offs because both variables may respond to weather factors such as precipitation in opposite ways (Knops et al. 2007).

In this current issue of *Tree Physiology*, Shestakova et al. (2024) investigate the responses of carbon source–sink processes to environmental constraints along climatic and productive gradients by combining long-term series of reproductive effort, radial growth and tree-ring isotope composition. They explore these issues on stone pine (*Pinus pinea* L.) stands of the Spanish Northern Plateau, where drought stress has been found to be the major climate driver of the performance of this Mediterranean conifer (Natalini et al. 2016, Calama et al. 2020). The stone pine is a gymnosperm species with large reproductive costs and masting behaviour, whose reproductive cycle extends over 4 years (Calama et al. 2008). The authors capitalized on the fact that nutlike edible seeds of stone pine are economically valuable, so there are relatively long series of cone yield at stand scale (Mutke et al. 2005). It is, hence, a suitable study system for unravelling the mechanisms controlling sink–source dynamics under climate warming scenarios.

Shestakova et al. (2024) provide evidence that a climate-driven shift from sink-limited to source- and sink-co-limited reproduction has occurred in recent decades, as shown by increasing interactive effects between climatic conditions during emergence and kernel filling driving cone yield. The authors argue that this may be the result of diminishing carbon resources as a consequence of drought stress and note the consequences for growth in view of the preferential allocation to storage and reproduction under carbon limitation (Wiley et al. 2017, Huang et al. 2021). Nevertheless, no evidence of competition for carbon resources between reproduction and radial growth was found, as both sink activities were positively and consistently associated throughout the study period (Shestakova et al. 2024), in accordance with previous research on the species (Mutke et al. 2005, Garcia-Barreda et al. 2021). However, caution is required before drawing such robust conclusions, as the statistical treatment of the data, such as detrending, together with secondary associations in other time lags (i.e., lagged 2 years) than the ones discussed (i.e., lagged 1 and 3 years) may imply different nuances in the competition between carbon sinks.

One of the main strengths of the Shestakova et al. (2024) research lies in the combination of structural (radial growth and reproduction) and functional (tree-ring carbon and oxygen isotopes, Δ^13^C and δ^18^O) traits, which has rarely been assessed when analyzing linkages between growth and reproduction (Ryan et al. 2018). This novel approach allows the authors to identify (i) climate warming as major driver of changes in reproductive source–sink dynamics and (ii) increasing reliance on fresh assimilates of reproduction, considering the regional positive associations involving Δ^13^C and cone yield at different time lags (Shestakova et al. 2024). This study is, therefore, based on the assumption that the dynamics of competing carbon sinks can be inferred from carbon and oxygen isotopic signals of tree rings (Gessler et al. 2014, Andreu-Hayles et al. 2022), as long as carbon acquisition and meristematic activity depend on similar environmental factors (Gessler and Ferrio 2022). The authors claim that the strong negative correlation between Δ^13^C and δ^18^O indicates that carbon assimilation is determined by stomatal regulation as stated by the dual-isotope approach (Scheidegger et al. 2000, Roden and Farquhar 2012). However, the interpretation of isotopic signals in wood is a challenging task because of the complexity of processes that control them. For instance, in addition to stomatal conductance, variations in source water δ^18^O composition (Sarris et al. 2013), mesophyll conductance (Ferrio et al. 2012) or exchange between organic oxygen and xylem water oxygen during cellulose synthesis (Gessler et al. 2009) have been found to influence tree-ring δ^18^O. Hence, caution is needed when analyzing Δ^13^C and δ^18^O series of tree rings.

Another potential shortcoming of the study of Shestakova et al. (2024) is the difference in the scales at which reproduction and radial growth are addressed. Spatial synchrony in reproductive effort of mast-seeding tree species is temporally variable: homogeneous in years with large seed crops (i.e., masting years), and heterogeneous during non-masting years (Koenig et al. 2003). As such, cone yield at the stand level may not accurately reflect tree- or branch-level processes such as cone or seed production as measured by tree-ring width and isotopes. For instance, branches may provide most of the carbon needed to produce the fruits they hold (Alla et al. 2012), and fruit production mostly relied on current photoassimilates (and not on old carbon stores) in three masting, temperate tree species (Hoch et al. 2013). Undoubtedly, future studies would benefit from long time series of individual-level data sets (Herrera 1998), which, however, pose logistical challenges in closed-canopy stands and mixed forests. Furthermore, radial growth is largely driven by cell expansion, which is relatively inexpensive in terms of carbon, but highly water-demanding because of its sensitivity to turgor changes (Körner 2015). Therefore, a density-weighted basal area increment would better reflect the actual carbon investment in radial growth rather than tree-ring width measurements.

Our knowledge of trees’ carbon allocation and use still needs more evidence to be conclusive. However, the study of Shestakova et al. (2023) is a step forward in understanding the source–sink dynamics of reproduction under the ongoing climate warming. Deciphering patterns of carbon allocation to radial growth and reproduction is highly relevant because of their linkages with key processes of forest functioning including competitive ability or natural regeneration. Here we argue that whilst such studies give us an important starting point, they would gain support by addressing the existing caveats (more robust stable isotope interpretation, spatial and temporal scales of proxies, and more reliable proxies of carbon investment on radial growth). Further studies exploring seasonal dynamics of non-structural carbohydrates and nutrients would additionally help to identify patterns of carbon allocation and nutrient use so as to gain a deeper insight to correlative approaches (Martínez-Vilalta et al. 2016). In the light of the results from the novel and stimulating research published by Shestakova et al. (2023) it remains to be tested in other forest systems and tree species encompassing a wide range of reproductive cycles and costs.

## Conflict of interest

None declared.

## Funding

This research was funded by the Science and Innovation Ministry (project TED2021-129770B-C21). E.G.A. is supported by the Spanish National Research Council (PIE-20223AT003).

## Data availability statemen

No new data or materials were created or analyzed in this study. Data sharing is not applicable to this article.
